# Climate change adaptation with limited resources: adaptive capacity and action in small- and medium-sized municipalities

**DOI:** 10.1007/s10668-023-02999-3

**Published:** 2023-02-09

**Authors:** Dennis Fila, Hartmut Fünfgeld, Heindriken Dahlmann

**Affiliations:** Institute for Environmental Social Sciences and Geography, University of Freiburg, Schreiberstraße 20, 79098 Freiburg, Germany

**Keywords:** Climate change adaptation, Small-sized municipalities, Medium-sized municipalities, Barriers, Capacities, Government, Governance

## Abstract

Administrations in small- and medium-sized municipalities (SMM) are confronted with the impacts of climate change while having inadequate resources to adapt. In order to establish the current state of research on climate change adaptation in SMM, a systematic literature review was conducted. Using reported SMM adaptation in the peer-reviewed literature as our data base, we documented 115 adaptation initiatives between 2015 and 2021 matching our criteria, with substantial geographical and thematic differences. The qualitative analysis of highly relevant articles has shown that the specific understanding about the challenges and barriers of climate change adaptation in SMM remains limited. We highlight recent key trends and challenges and conclude by offering a refined research agenda for addressing identified knowledge gaps as well as key barriers in relation to SMM adaptation.

## Introduction

The climate change induced rise in extreme weather events such as floodings, or heat waves have raised the need for political and administrative actors across scales to develop strategies for adaptation. Over the past two decades, the scientific discourse on implementing local climate change adaptation (CCA) has grown substantially. The local spatial scale has been deemed particularly important for adaptation because first, the climate change impacts materialize most tangibly in a given local context and second, local institutions are often considered best placed for adapting through context-based measures and strategies (Boehnke et al., 2019; Lee et al., 2020). We understand adaptive capacities at the local level as a range of processes and factors that support systems to adapt to climate change and other types of environmental changes (Mortreux & Barnett, 2017).

Social science discourses on local adaptation have so far centered around the importance of municipal governments (Measham et al., 2011; Pasquini et al., 2015) and the significance of the local level of administration, planning, and decision-making within a multi-level climate change adaptation governance context (see, among others, Adger, 2005; Mukheibir et al., 2013; Lioubimtseva and Da Cunha, 2020). Much of this scholarly work on municipal adaptation focuses on adaptation in bigger cities (Birkmann et al., 2014; Reckien et al., 2018). This is not surprising, given that larger cities have been the frontrunners of municipal and urban adaptation, supported by relatively strong fiscal capacities that include targeted international funding programs, such as the 100 resilient cities campaign from 2013 to 2019 (Resilient Cities Network, 2021). Comparatively little research on adaptation and adaptive capacity has been conducted in small- and medium-sized municipalities (SMM) (Bausch & Koziol, 2020; Hoppe et al., 2016). SMM are mostly located in rural areas. Despite ongoing urbanization, 3.4 billion inhabitants or close to half of the global population (44.2%) still lived in non-urban areas in 2020 (World Bank, 2020).

Given this and the specific circumstances SMM face with advancing adaptation, the urgency to examine adaptation in the municipalities of this size is evident. SMM differ fundamentally from larger cities due to their economic, political, environmental, and social characteristics and are therefore exposed differently to the consequences of climate change. Correspondingly, challenges, barriers, and opportunities to increase adaptive capacities of local governments in SMM remain poorly understood, calling for locally contextualized adaptation research in these municipalities. In this paper, we aim to contribute to closing this gap by taking stock of existing literature on adaptation in SMM with a conceptual emphasis on adaptive capacity. Our review is guided by the hypothesis that the lower administrative adaptive capacities in SMM also result in less effective climate change adaptation in the respective municipalities. More generally, this review addresses the question to what extent adaptive capacities in institutions and administrations are represented in previous studies on climate change adaptation in SMM and in which dimensions they differ from larger cities.

The definition of SMM is complex and contingent on a variety of country-specific factors. Population size is the most common parameter used for delineating small from large cities. However, in scientific publications, vastly different definitions regarding population size exist (Hamin et al., 2014; Paterson et al., 2017) of which some are grounded in national stipulations enshrined in national planning law. In Germany, municipalities with more than 100,000 residents are considered large cities in spatial planning law, while cities with fewer than 20,000 inhabitants are referred to as ‘small.’ For this review, this German size definition will be used, with the modification that the upper limit for the inclusion of cases has been increased to municipalities with 200,000 inhabitants to better reflect international thresholds of population size. Similarly depending on local nomenclature, research on SMM commonly focuses on either districts, cities, counties, or municipalities. In the context of this review, the term municipality is utilized as an umbrella term that also incorporates the other local territorial units mentioned above.

This review consists of four parts: the first part includes a detailed description of the methodology used for the systematic review and includes a discussion of the limitations of the obtained data. In the second part, this is followed by a geographical overview of the studies considered relevant, also identifying spatial clusters and gaps. The third section displays an overview of the topics in CCA identified in the studies. Based on the most informative cases identified, section four debates the main developments and challenges of adaptation and adaptation capacity building in SMM. Drawing on the discussed research and the knowledge gaps, a research agenda for adaptation in SMM is developed in this final section of the paper.

## Methodology

To identify studies about adaptation in SMM, a systematic review was carried out. The methodological approach is based on systematic literature reviews on CCA and public participation (Hügel & Davies, 2020) and adaptive pathways (Bosomworth & Gaillard, 2019) and was supplemented by the methodological approach of Cerchione and Esposito (2016), which is presented below.

**Fig. 1:**
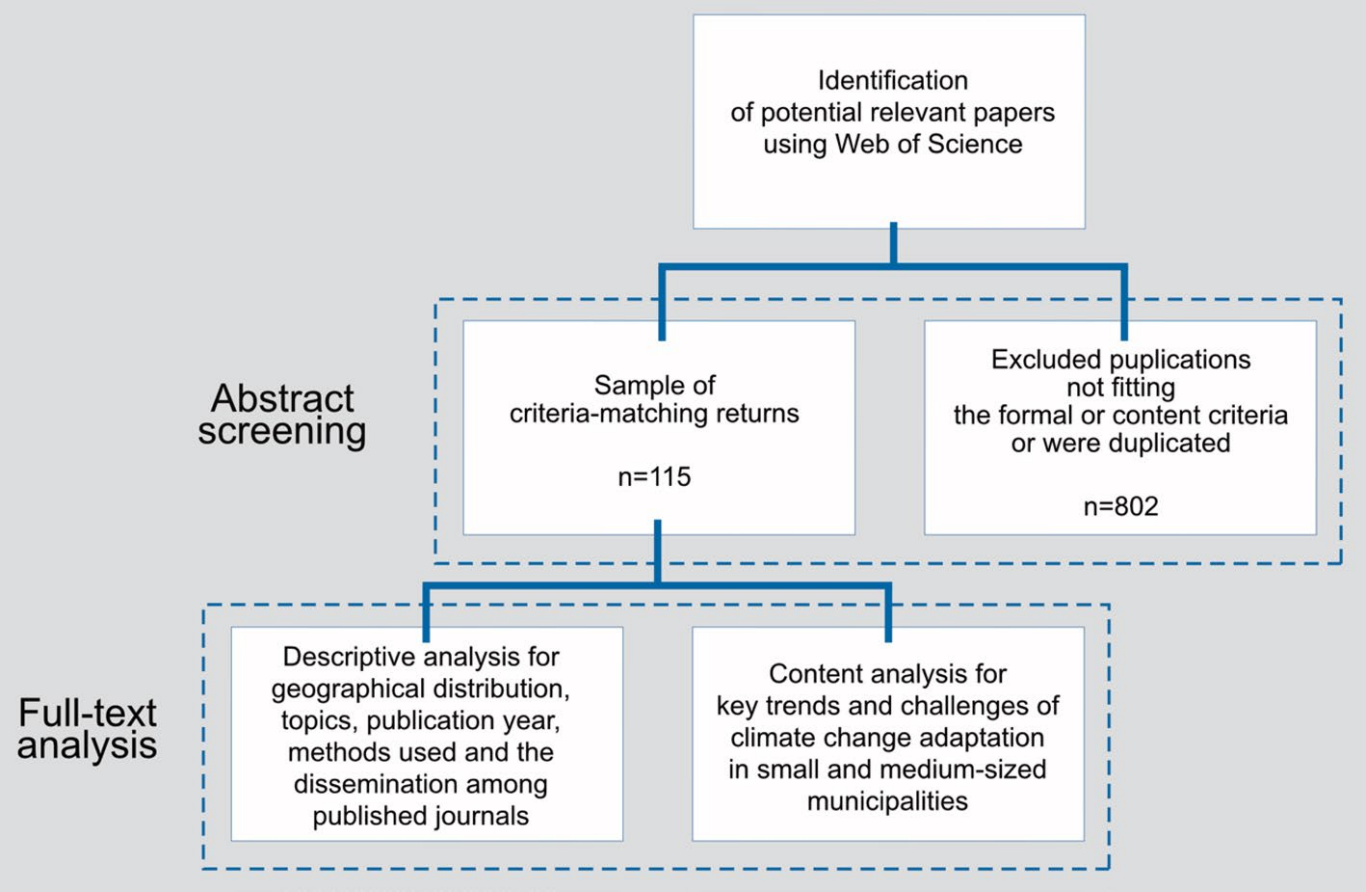
Methodological approach of the systematic review

The first phase of the material comprehensive search (ibid.) consisted of the definition and evaluation of key word search terms as well as choosing the Web of Science database because it produced the most comprehensive results. English-language peer-reviewed literature of the last six years (January 2015 until December 2021) was used as data source. To identify the broadest possible spectrum of studies, this review used four pre-defined subsets of keyword combinations (see Table 1). The systematic search was conducted for abstract, title, and keywords and produced 917 potentially relevant articles (Fig. 1).

**Table 1:** Keyword search in the Web of Science database and screening steps

Subset	Search terms	Returns	Criteria-matching returns	After de-duplication
1	Climate change adaptation AND (municipal* OR local government)	321	67	67
2	Climate change adaptation AND community-based	149	34	28
3	Climate change adaptation AND (small-size* OR medium-size*)	27	4	1
4	Climate change adaptation AND rural	420	28	19
Total		917	133	115

In the next step, each abstract was read to determine the relevance of the paper using a previously defined collection of exclusion and inclusion criteria (Table 1 in the supplementary material). The selection was based on the content of the relevant publications identified in advance and the specific epistemic interest (see chapter 1). The criteria were established before the literature search and were not changed during the ensuing process. In addition to the already mentioned formal criteria concerning the peer-review status, data range, and language, five content-related criteria were defined. Many of the excluded studies predominantly engaged with stakeholders and governments at regional and national levels. In addition, many cases dealt with technical aspects of climate change adaptation, adaptation in natural systems, or only with non-governmental actors, which also resulted in their exclusion. After performing the de-duplication and applying the inclusion and exclusion criteria mentioned, more than 87% of the studies were excluded. More than half of the 115 remaining publications can be assigned to the first search subset, while only one relevant study was identified in the third subset (see Table 1).

**Fig. 2:**
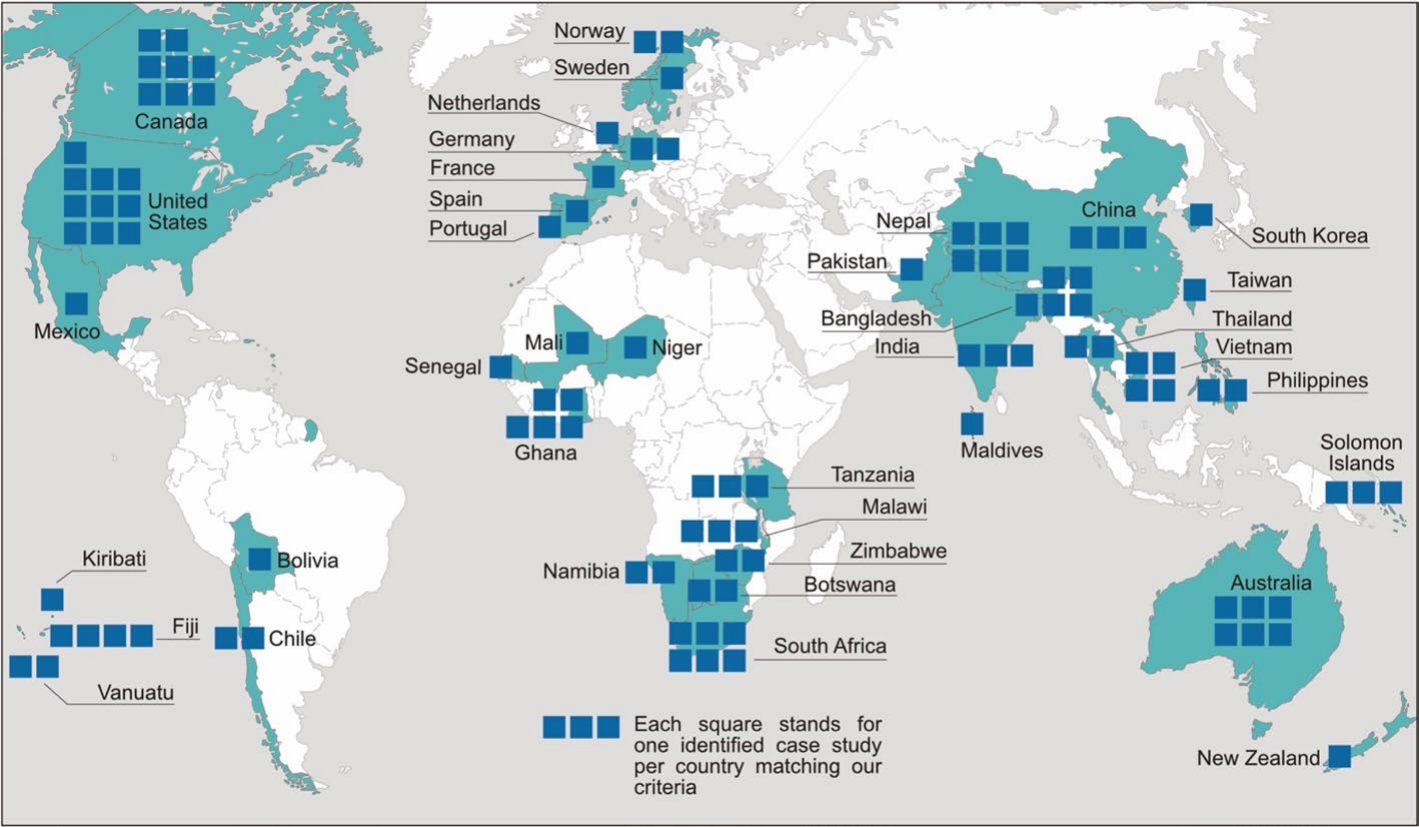
Regional distribution of the identified studies. Nine case studies compared case studies in different countries and were not including in this figure (see supplementary material). Total: 106 cases in 39 countries

Following this first phase, a content analysis of the selected articles was performed in the second phase to obtain an overview of the corpus. Documents were analyzed to map their geographical distribution. Each identified study was assigned to a country following the UN members list. Studies containing a comparison between different countries were labeled as *comparative.* The abstracts of the included studies were scanned for their main research focus. The sectoral risks defined by the (IPCC, 2014) were used as topics for this categorization (see Fig. 2 in Sect. 3). The outcomes of the descriptive analysis are shown in the third section. For the final step of the content analysis, in Sect. 4 all included articles are evaluated regarding their elaboration on key trends adaptation in SMM and the associated research gaps.

The chosen approach of data collection and analysis harbors some methodological limitations. Because only peer-reviewed papers were used for this systematic review, care is vital when reading the results. Due to the exclusion of non-peer-reviewed publications, the review is biased as it does not cover potentially important grey literature or other excluded publications, such as publications with applied and transdisciplinary focus that may have opened important practice-based insights. Due to language limitations, non-English publications were excluded completely, which can be assumed to have substantially affected the results. For example, as the result in section three shows, almost no cases of adaptation in SMM in Latin America were identified. This may be because many relevant publications for this region are published in Spanish or Portuguese. While four different keyword combinations were used with the intention to identify a variety of applicable cases, it cannot be ruled out that further combinations would have resulted in additional publications.

## Descriptive results of the systematic review

Figure 2 illustrates the highly uneven geographic dissemination of identified cases of SMM adaptation. Most of the cases were found in North America. The largest number of identified studies on adaptation in SMM stems from the USA, with a clear focus on coastal adaptation, mainly due to increasing hurricanes (see for example, Fischer, 2018; Jurjonas et al., 2020), and permafrost as well as livelihoods in Alaska (Birchall & Bonnett, 2020; Loring et al., 2016; Ristroph, 2021). Canada also stands out with a high number of identified case studies, with the main focus of governmental aspects of adaptation (Beaulieu et al., 2016; Reeder et al., 2020). In contrast, in Latin America, significantly fewer cases referring to adaptation in SMM emerged. However, based on this review it cannot be ascertained whether this is related to lower adaptation activity, limited scientific coverage, or a result of other aspects of the review methodology, as discussed above.

In relation to overall population size, a relatively large number of SMM adaptation case studies study the South Pacific parts of Oceania. These can be linked to adaptation action against sea level rise and increasing extreme weather events. The area is particularly exposed to those impacts due to low-lying island settlements. Case studies from this region deal with the relocation of whole communities (Fiji: Bertana, 2020), adaptation to extreme weather-like tropical cyclones (Solomon Islands: Ha'apio et al., 2019, Vanuatu: Le Dé et al., 2018), and community-based adaptation (see, among other, Fiji: Remling & Veitayaki, 2016; Kiribati: Piggott-McKellar et al., 2020; Solomon Islands: Basel et al., 2020; Vanuatu: Westoby et al., 2020). In addition to the coverage of adaptation in South Pacific island countries, a disproportionately large number study Australian SMM. The identified Australian case studies in SMM adaptation mainly deal with coastal management (Frohlich et al., 2019; McNamara et al., 2020; O’Donnell, 2019), with indigenous adaptation (Nursey-Bray & Palmer, 2018), and with adaptation governance on the local level (McClure & Baker, 2018; Torabi et al., 2017).

Another large cluster of adaptation studies focuses on SMM in Southeast, East, and South Asia. Countries with particularly high exposure in rural areas are frequently covered by case studies, such as Nepal, China, Bangladesh, and Vietnam. SMM adaptation case studies in this region mainly address agricultural adaptation and local policies for food supply (see, among others, Bangladesh: Karim & Thiel, 2017; China: Xu & Findlay, 2019; Nepal: Maharjan, 2020; Vietnam: Halbherr et al., 2021). Other foci are freshwater ecosystems (India: Sen & Kansal, 2019; Pakistan: Qazlbash et al., 2021), disaster risk management (see, among others, China: Wang et al., 2019; Philippines: Dujardin et al., 2018; Vietnam: Christoplos et al., 2017), and sustainable livelihoods (see, among others, Bangladesh: Paprocki, 2018; Morsalin & Islam, 2021; Vietnam: Mabon et al., 2020).

Furthermore, a cluster of studies on SMM adaptation focuses on Sub-Saharan Africa, especially Eastern and Southern Africa. Key foci of SMM adaptation research in this region are: drought and water management issues (Namibia: Davies et al., 2020; Tanzania: Velempini et al., 2018; Zimbabwe: Mubaya & Mafongoya, 2017; Mugambiwa & Makhubele, 2021); agriculture (Botswana: Mogomotsi et al., 2020; South Africa: Ziervogel et al., 2017); and—especially in South Africa—sustainable livelihoods: see, among others, Ziervogel, 2019; Spires & Shackleton, 2018). Eight studies have focused West African SMM with foci on governance and/or gender (Ghana: Garcia et al., 2021; Musah-Surugu et al., 2019; Mali: Totin et al., 2021; Senegal: Vedeld et al., 2016; Niger: Tabbo & Amadou, 2017) and droughts (Ghana: Yomo et al., 2020). No such adaptation studies deal with MENA countries, although the climate change-related impacts will be particularly severe in this region and bear upon a large population (IPCC, 2014).

The number of documented studies on SMM adaptation in Europe is also exceptionally low and mostly focuses on Western and Northern Europe. Only two case studies are addressing individual topics: human health (Hernandez et al., 2018) and coastal regions (France: Rocle & Salles, 2018). The other European studies deal with cross-cutting aspects of adaptation governance, such as the particular situation of SMM (The Netherlands: Hoppe et al., 2016; Germany: Bausch & Koziol, 2020; Huber & Dunst, 2021); Portugal: (Campos et al., 2017), adaptation in a multi-level-setting (Norway: Hauge et al., 2019; Orderud & Naustdalslid, 2020), and citizen–municipality interactions (Sweden: Brink & Wamsler, 2018).

In summary, the geographical distribution of scientific studies on SMM adaptation shows that adaptation is mostly researched in countries and areas that are already severely influenced by the impacts of climate change and that have significant research capacity in this area. This includes regions that experience severe extreme weather events, or are at risk from rising sea levels (see, among other, USA, and Oceania); droughts and water insecurity (e.g., southern areas of Africa or Eastern parts of Asia); and food insecurity. At the same time, our review highlights that some regions are overrepresented in English-language adaptation research, while other regions such as Europe and Latin America are underrepresented in relation to the number of inhabitants. Some regions like MENA or Eastern Europe are not covered at all by the current literature on adaptation in SMM from the last five years.

**Fig. 3:**
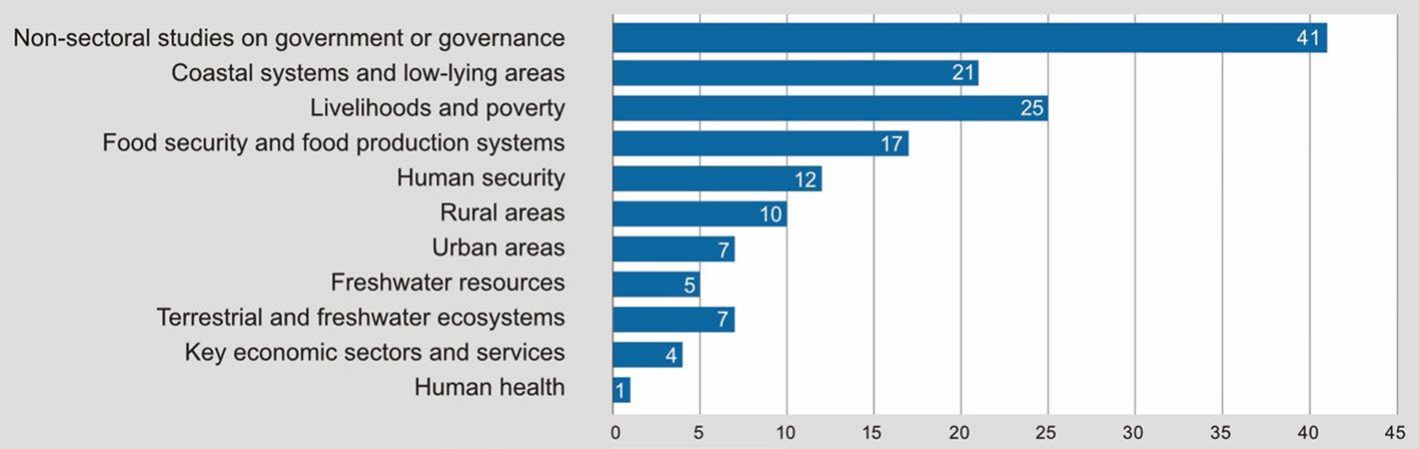
Frequency of topics in the identified studies, 115 total peer-reviewed articles, multiple assignment of topics to single case studies possible

The general overview of the assigned topics (Fig. 3) shows that a large part of the literature included in the corpus mainly focuses on government or governance (*n* = 41) as a cross-cutting issue and less on individual topics. This trend is particularly evident in the literature on community-based adaptation. Livelihoods and poverty (*n* = 25), coastal systems and low-lying areas (*n* = 21), food security (*n* = 17), and human security such as threads by hurricanes (*n* = 12) were the most common topics. Less frequent were aspects of human health (*n* = 1) and questions of key economic sectors and services (excluding agriculture) (*n* = 4).

**Fig. 4:**
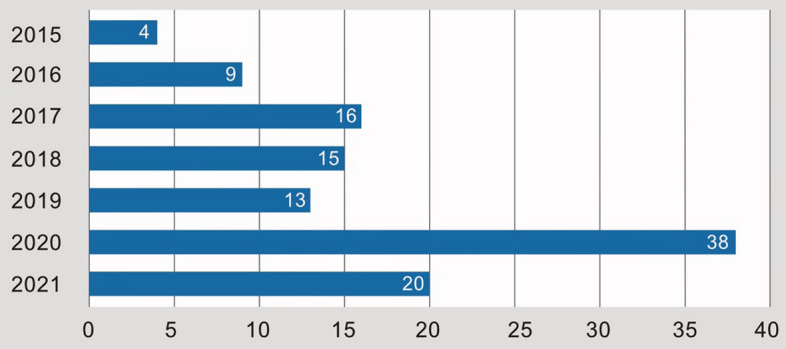
Distribution of included publications by year

The temporal distribution of publications by publication date shown in Fig. 4 indicates one third of the publications included in this systematic review were published in 2020. Between 2017 and 2019, a total of 44 publications were published, only six more than in the year 2020, with the share of publications from 2015 to 2016 being particularly low. There is a significant drop again in 2021 with only 20 publications what might be explained by the consequences of the Covid-19-Pandemic and the subsequent troubles for conducting field work.

**Fig. 5:**
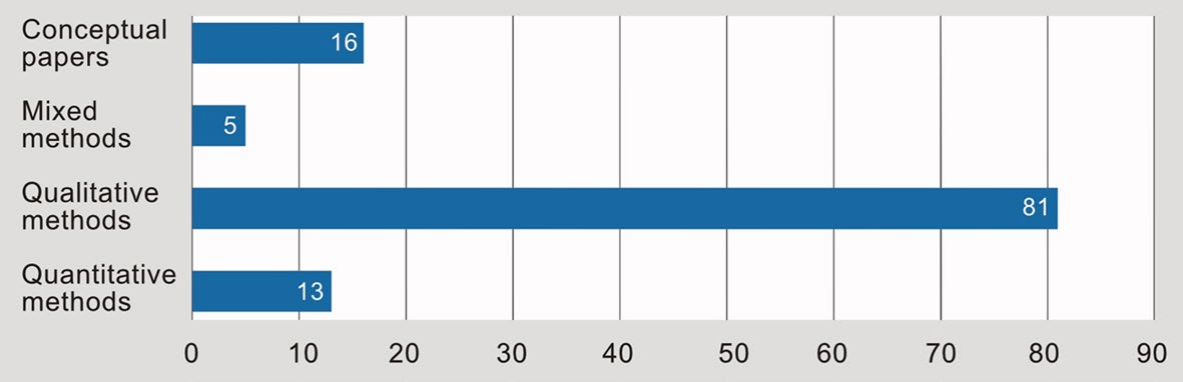
Distribution of the 115 included studies by methodology

A closer look at the methods used (Fig. 5) in the individual cases on climate change adaptation in SMM shows that an overwhelming part (*n* = 81) focuses on qualitative methods—especially expert interviews, focus groups, and participant observation. A clear literature gap is visible in the use of quantitative methods in this field. In addition, there are about five studies that combined qualitative and quantitative methods. About 16 conceptual papers are included, which are not based on primary empirical data but evaluate secondary data and information.

Table 2 shows that case studies on adaptation in SMM are predominantly published in journals that do not focus on individual journal subject areas but instead cover a broader range of topics. The identified five subject areas ‘geography, development and planning,’ ‘health sciences,’ ‘sociology and political sciences,’ ‘agricultural and biological sciences,’ and ‘environmental physical sciences’ show that climate change adaptation cuts across almost all subject areas of science and that therefore an inter- and transdisciplinary approach is necessary when dealing within this context. Nevertheless, the frequency of identified journal subject areas suggests that the case studies in this review are published in journals with a focus on ‘geography, development and planning,’ ‘sociology and political sciences,’ or ‘environmental physical sciences.’ Most frequently, studies were published in the journals ‘climate and development’ (*n* = 9), ‘sustainability’ and ‘regional environmental change’ (*n* = 6), and ‘environment and science policy’ (*n* = 5).

**Table 2:** Dissemination of the case studies among journals

Journal	Total	Journal subject area				
		Geography and planning	Health sciences	Sociology and politicology	Agricultural and biological sciences	Environmental sciences
African geographical review	1	x				x
Ambio	2	x		x		x
Annals of the American association of geographers	1	x				x
Asia pacific viewpoint	1			x		
Australian journal of agricultural and resource economics	1				x	x
Bulletin of Latin American research	1			x		
Climate and development	9	x				x
Climate policy	1			x		x
Climate risk management	4	x				
Climatic Change	4	x				x
Development Southern Africa	1	x				
Disasters	2					x
Disaster prevention and management	1	x				x
Environment, development and sustainability	2	x				x
Environment and planning C: politics and space	1	x		x		
Environmental hazards	3	x				x
Environmental justice	1	x				
Environmental management	1	x		x		
Environmental research	1		x			x
Environmental research letters	1	x				x
Environmental science and policy	5	x		x		
Geographical research	1	x				
GeoJournal	1	x				
Heliyon	1	x	x	x	x	x
Human ecology	1	x	x			
International journal of climate change strategies and management	2	x				x
International journal of conservation science	1				x	x
International journal of disaster risk reduction	2	x		x		x
International journal of sustainable development and world ecology	1	x				x
Journal of environmental studies and sciences	2	x				
Jamba (Potchefstroom, South Africa)	3	x				x
Journal of environmental management	3	x			x	X
Journal of environmental planning and management	1	x		x		
Journal of rural and community development	1	x		x		
Journal of water and climate change	1	x				x
Land use policy	4					
Landscape and urban planning	2					
Local environment	4	x		x		
Marine pollution bulletin	1		x		x	x
Mitigation and adaptation strategies for global change	1	x		x		x
Natural hazards	2					x
Nature climate change	1				x	x
Ocean and coastal management	4	x			x	x
Planning theory and practice	1	x		x		
Policy sciences	2			x		
Public performance and management review	1			x		
Regional environmental change	6	x				x
Risk, hazards and crisis in public policy	1	x		x		x
Spatial research and planning	1	x		x		
Sustainable development	1	x		x		x
Sustainability science	1	x				x
Sustainability	6	x	x	x		x
Urban climate	2	x				x
Urban policy and research	1	x		x		
Weather, climate, and society	3	x		x		x
WIREs climate change	1	x				x
World development	2	x		x		

## Content analysis and discussion of key trends and challenges

### Barriers of adaptation in SMM

With limited institutional capacities resulting from the size of municipal governments, structural barriers are particularly present in SMM. The most striking barrier referred to in the reviewed studies is the lower number of employees in comparison with larger municipalities and the lack of adequate internal organization to enable and progress adaptation (Campos et al., 2017; Hoppe et al., 2016). Where staff capacity is lacking, adaptation planning is likely to be constrained (Birchall & Bonnett, 2020).

Larger municipalities have a higher number of individual departments, which are also more specialized. Having relatively fewer departments in SMM on the other hand means that these tend to have to deal with more diverse tasks and are therefore less able to also focus on adaptation as additional independent task (ibid.). In the German state of Bavaria, a survey among municipalities with less than 20,000 inhabitant showed that most of the very small municipalities (less than 2,000 inhabitants) addressed adaptation almost exclusivly within their elected municipal councils (Bausch & Koziol, 2020). Municipalities between 10,000 and 20,000 inhabitants, on the other hand, developed strategies within the muncipal administration (87%) and jointly with civil society (57%), in addition to also addressing the topic in their elected council (61%).

Similar differences also became apparent with regard to implementing adaptation strategies. According to the survey, the accountability for adaptation in very small municipalities lies largely with the mayors (83%) and only to a small extent with the respective administrations (33%), while in municipalities between 10,000 and 20,000 inhabitants it is exactly the opposite. (ibid.). Compared to larger cities, SMM rarely have a holistic approach to climate change adaptation, but instead focus more on individual topics, which is particularly eminent in very small municipalities (ibid.). In addition, capacity constraints also lead many SMM to respond reactively to climate-related disasters rather than developing measures proactively (Orderud & Naustdalslid, 2020).

To varying degrees, the studies show that adaptation policy is slowed down by fiscal and political influence, depending on local context (McClure & Baker, 2018). Smaller municipalities have limited capacities in terms of financial and human resources, which limits the possibilities for adaptation measures. Relatively low financial capacities mostly result from a small tax base and a less diversified economy and thus lead to a greater dependency on external funding and investments (Birchall & Bonnett, 2020; Pasquini, 2019).

A major challenge within adaptation planning in SMM is the rather long-term temporal scope of the planning effort. For adaptation to take on strategic qualities, time horizons covering the coming decades need to be considered. Such a long-term view is not inherent in typical measures-driven adaptation plans: politically, long-term planning at the scale of SMM is limited by electoral cycles and prevalent short-termism among political decision-makers (Campos et al., 2017). This also results in ‘project-based’ adaptation planning, where adaptation measures developed as part of initial adaptation plans are not carried forward into further iterations of a plan (see, for example, Birchall & Bonnett, 2020).

Among the most frequently mentioned barriers to adaptation in SMM were those which refer to the policy framework of adaptation governance. Local adpatation is embedded in specific, mostly hierarchical systems of multi-level governance, in which different jurisdictions prevail at different levels. Several of the reviewed studies conclude that fragmented power structures across scales and unclear assignment of legal responsibilities to local governments impede effective context-based adaptation planning, especially for SMM with limited capacities. Although the local administration is frequently identified as the level of government that, due to experiencing direct impacts of climate change and having direct access to local knowledge, can most effectively develop measures for adaptation, in many countries centralized governance structures hinder such locally led adaptation (Soanes et. al., 2021), for example, through a plethora of poorly aligned political and strategic priorities, strict top–bottom regulations, lack of coordination, or the time delay of measures and authorization (Clissold et al., 2020; Davies et al., 2020). Campos et al. (2017) showed that Portunguese cities with over 50,000 inhabitants had very extensive data bases for adaptation planning, while such data were scarce or non-existent in smaller municipalities. The authors explain the knowledge gap by a lack of often expensive downscaled regional data and reports. In addition, SMM in peripheral areas often struggle with access to appropiate technologies and services when compared to their central counterparts (Clissold et al., 2020, Islam & Nursey-Bray, 2017).

Such information deficiency also results in structural financial disadvantage: in their case study in The Netherlands, Hoppe et al. (2016) demonstrate that larger cities benefit disproportionately from national subsidies, while SMM have been allocated such funds less often due to lower knowledge of programs, smaller capacities in the application process, and the lack of a ‘critical mass.’ In Namibia, national governments often try to initiate climate change adaptation processes on a local scale throughout a centralized ‘one-size-fits-all’ approach lacking a municpal perspective, which leads to maladaptations (Davies et al., 2020).

Not only the cooperation with higher levels of government but also with other cities and municipalities in networks differs substantially between larger cities and SMM. Many of the transnational municipal networks for adapation have a heterogeneous representation of members from the Global North and Global South. Nonetheless, these are primarily designed to benefit and involve larger cities. Correspondingly, SMM are represented in fewer transnational municipal networks (Pasquini, 2019). Where municipalities are members of such networks, SMM benefit from the knowledge exchange and better access to funding oppurtunities, as evidenced by studies from Portugal and Norway (Campos et al., 2017; Orderud & Naustdalslid, 2020).

At a local scale, SMM also face barriers with regard to devising participatory processes for enabling adaptation. As a result of lower institutional capacities, SMM are often dependent on voluntary adaptation action of local organizations and community members. In the context of water point committees in Namibia (Davies et al., 2020), volunteers were only able to perform committee-related tasks to a limited extent due to their need for engaging in daily activities that sustain their livelihood. In addition, community members may become stuck in what Davies et al. (2020) called a ‘vulnerability trap’: without targeted investments in rural communities decentralized adaptation at the local scale can lead to maladaptation and entrenched vulnerabilities due to local capacity constraints and knowledge mismatches. On the other hand, dependency problems can arise through external funding because of conflicting goals and strategies of the funding agencies (ibid.). This, in turn, can impede self-sufficient adaptation in societies and local governments and instead create a dependency on external investments (ibid.). In any case, effective participation in adaptataion is challenging to achieve for SMM. A case from Bangladesh emphasizes that the local government only facilitated citizen and community participation in SMM adaptation as part of the execution of measures but not in the other stages of the adaptation progression (Islam & Nursey-Bray, 2017). However, participation of such actors in various phases of the adaptation processes is of great importance, as these actors may hold important municpal knowledge to interpret the localized effects of climate change and to plan and help carry out possible adaptation measures (Hoppe et al., 2016; Islam & Nursey-Bray, 2017; Pasquini, 2019). Often, especially in SMM, technical adaptation solutions are sought, even though they hardly adress neither the root causes nor measures and strategies that could increase community capacities (ibid.).

In addition to the above-mentioned institutional barriers, cognitive barriers also have a huge role in SMM. The perception of risk, beliefs, and goals in relation to adaptation depends very much on local characteristics, such as cultural determinants and the balance of power between actors, which can influence the acceptance of adaptation measures and strategies (Hoppe et al., 2016, Islam & Nursey-Bray, 2017). In many countries and especially in rural areas, due to poor state and local structures or capacities, non-governmental organizations are the main initiators and implementers of climate change adaptation. Since they often work with higher levels of government and single stakeholders, they can unintentionally neglect the municipal paths for sociatal action (Davies et al., 2020) and thus sideline SMM.

### Adaptive capacity building in SMM

As established through the examples, SMM are characterized by an institutional context of structurally limited adaptive capacity. Consequently, and in spite of such limitations, individuals can play particularly important roles in adaptation processes. Staff members with either formal responsibility for, or with a professional or disciplinary affinity to, climate change adaptation frequently act as pioneers or champions. They take on the role of a knowledge facilitator and thus generate awareness among colleagues and external actors they are in touch with (Bausch & Koziol, 2020; Dale et al., 2020; Pasquini et al., 2015). If they are supported by leadership, they can become key drivers for adaptation, thus moving from a pioneering role to one of consolidating and institutionalizing adaptation efforts. Such ‘pioneering’ also plays a significant role in inter-organizational collaboration for adaptation in SMM (Bausch & Koziol, 2020). Across SMM, progressing adaptation efforts often rely on individual local government actors that take on leadership, for example, by attracting third-party funding or by initiating regional cooperation on climate change adaptation (Fünfgeld & Robertson, 2016). As a result of their efforts, such pioneers can make resources available—either from internal budgets or external sources—to hire dedicated staff (e.g., climate protection managers co-funded by the Federal Government in the German context), who then may also be in a position to support neighboring or smaller municipalities in their vicinity (Bausch & Koziol, 2020). Through such specialization, especially when combined with strong leadership support within the organization and content-focused networking across organizational boundaries, municipalities can develop their administrative and knowledge capacity about climate change adaptation from within. However, it is not surprising, as Bausch and Koziol (ibid.) point out, that such pioneering, cross-municipal facilitators are mainly to be found in municipalities with over 5000 inhabitants, where necessary institutional support is available.

As highlighted by Hoppe et al. (2016), adaptation officials need important skills to be able to act as policy entrepreneurs and ‘manage up’ to bring adaptation to the policy agenda. Gradual built-up of support for adaptation policy agendas can be critical for when ‘windows of opportunity’ open up, where such policy entrepreneurs can play their trumps and activate adaptation planning processes that were prepared in advance (ibid.). For individuals to develop and use such skills when the opportunity arises requires them to self-identify as an activist-type bureaucrat with a desire to change existing institutional processes and structures (ibid.).

Where institutional support is not available, pioneering initiatives in adaptation are likely to be short-lived if they can emerge at all. Even where additional resources are made available for dedicated staff capacity, policy measures are necessary to support the development of institutional capacity for adaptation. Here, formal instruments of land use planning play a central role (Bausch & Koziol, 2020). Compulsory consideration of climate change in land use zoning and building controls can catalyze adaptation efforts, even in low-capacity contexts. Where such mandatory consideration exists, as it is the case in Germany, municipal administrators and decision-makers are forced to develop and evaluate draft land use plans in light of specific climate change risks, like flooding or wildfires. In many countries, planning overlays exist to demarcate areas where such considerations are mandatory and where they are not (e.g., in the UK, Germany, and Australia, for different hazards). By requiring consideration of both the climate footprint and climate change risks and vulnerabilities, land use planning thus provides critical opportunities for medium-term strategic adaptation, as such plans are typically legally binding for decades (Bausch & Koziol, 2020). Especially for SMM, institutional and financial support may be required following the introduction of mandatory climate change-sensitive land use planning, to assist municipalities with developing relevant expertise and compensate for increased planning costs (ibid.). Overlays, ordinances, and other formal planning tools also raise awareness among developers and residents (Birchall & Bonnett, 2020) while also running the risk of shifting the burden of adaptation to people already vulnerable to climate change-related impacts.

In the context of New Public Management and the prevailing paradigm of evidence-based policy making, decision-support tools, such as cost–benefit analysis (CBA) and cost-effectiveness analysis (CEA), have become major tools for evaluating adaptation measures *ex ante* (Campos et al., 2017; Hallegatte, 2009). However, SMM rarely have the capacity to conduct such analyses, nor do they have the funds to pay external consultants to do such work. Where CBA and CEA are considered, pooling of resources across SMM in a given region, or coordination of such analyses at district or regional level, can be options for overcoming capacity constraints at the local scale. Despite this substantial list of knowledge and capacity constraints identified in the recent literature, SMM may also benefit from locally contextualized positive dynamics that can enable local adaptation more readily than in larger cities, e.g., the fact local actors care about climate change impacts and show high levels of motivation to address climate change impacts (Pasquini, 2019). Not only local government officials in SMM but also residents may display greater personal identification with climate change adaptation, as their experience of climate change impacts may be more immediate when compared to citizens in larger urban centers (ibid.). Such cognitive and affective dimensions may positively influence and support climate change adaptation efforts in SMM (ibid.).

## Conclusion and further research agenda

This review of recent literature gave insights into the status of knowledge on adaptation in SMM. The geographical distribution of the identified cases shows that there is a spatially uneven coverage of local climate change adaptation in SMM. Recent studies on SMM adaptation were concentrated on some countries and regions, such as Oceania, North America, and Southern Africa, while Europe, the Middle East, and North Africa are clearly underrepresented in the identified scientific documents.

### Contribution to adaptation practice

The reviewed studies focused mainly on the barriers of adaptation in SMM. The case studies showed that formal adaptation of SMM is characterized—and heavily constrained—by limited resources. The most significant barriers to adaptation identified in SMM studies are limited financial and personnel capacities in municipal administrations (Campos et al., 2017; Hoppe et al., 2016). SMM climate change adaptation is characterized by diverse political and policy contexts and, as such, embedded in a multi-level governance structure that includes regional and national stakeholders with (potentially) conflicting goals and a high dependency on top-down streams of funding (Birchall & Bonnett, 2020; Pasquini, 2019). Compared to bigger cities, SMM have larger knowledge gaps of the likely local consequences of climate change and possible adaptation measures and strategies (Clissold et al., 2020, Islam & Nursey-Bray, 2017). Local structures of political power, economic, and financial capacities differ greatly across the identified studies and existing approaches to adaptation developed predominantly in larger cities and regions cannot be easily transferred to SMM (Hoppe et al., 2016, Islam & Nursey-Bray, 2017).

In addition to this plethora of intertwined barriers, some case studies highlighted how such barriers can be overcome and how SMM can increase their adaptive capacity. For example, SMM can profit from the structural framework of adaptation governance if higher level of governance supports municipalities with financial, administrative, and knowledge capacities without enforcing ‘one-size-fits-all’ approaches for adaptation. Instead, multi-level governance regimes can afford SMM the opportunity to use local knowledge and develop context-based solutions and strategies and thus enable effective local adaptation. SMM particularly benefit from the fact that there is a stronger network of local actors in smaller municipalities which enables better informal and formal sharing of ideas and strategies (Birchall & Bonnett, 2020; Pasquini, 2019).

### Contribution to theory and further research agenda

However, as indicated by the low number of studies identified that highlight how barriers of climate change adaptation planning can be overcome in SMM, there are still considerable knowledge gaps with regard to progressing and enabling climate change adaptation in SMM. To remedy this lack of solution-orientated knowledge, we suggest expanding current research efforts to address the following points:

1. Extending ‘stocktake’-type studies of local adaptation needs, processes, barriers, and enablers to currently underrepresented geographical areas, especially those in the rural periphery;
2. Examining the distinct responsibilities of key stakeholders in planning and implementing climate change adaptation in SMM and within corresponding multi-level governance contexts, including by drawing on comparative research across countries and constituencies;
3. Conducting quantitative and qualitative social research to distinguish more clearly the unique needs for climate change adaptation planning across large-, medium-, and small-sized municipalities, districts, and cities;
4. Continued and more nuanced case study research on how barriers in adaptation planning and adaptive capacity constraints have been overcome in institutional contexts with limited resources;
5. Conducting research into innovative pathways for holistic planning approaches for climate change adaptation that transcend sectoral and project-based adaptation efforts, including the development of culturally appropriate models for effective adaptation.

Addressing these, we hope, will contribute to a significantly better geographical coverage of adaptation planning and implementation outside of urban centers and the role of formal and informal actors in SMM contexts. From existing knowledge and the case studies, policy implications can only be derived to a limited extent, as the data and information collected in the studies identified as part of this review are largely highly context-specific and can therefore hardly be generalized. Furthermore, the focus of the identified studies was mostly on individual sectors and stakeholders in SMM, rather than on approaches that address climate change adaptation in its full breadth. During content analysis, it became clear that mostly qualitative methods are used within this context and that therefore a methodological bias is apparent. An inter- and transdisciplinary approach to such research seems not only desirable but necessary to enable learning and collaboration across disciplines and communities of practice.
